# Afoxolaner against fleas: immediate efficacy and resultant mortality after short exposure on dogs

**DOI:** 10.1051/parasite/2014045

**Published:** 2014-08-25

**Authors:** Frédéric Beugnet, Christa deVos, Julian Liebenberg, Lénaïg Halos, Josephus Fourie

**Affiliations:** 1 Merial S.A.S. 29 avenue Tony Garnier 69007 Lyon France; 2 ClinVet International, Universitas 9301 Bloemfontein, Free State South Africa

**Keywords:** *Ctenocephalides felis*, Dogs, Afoxolaner, Short exposure, Immediate killing, Induced mortality

## Abstract

The speed of efficacy of afoxolaner (NexGard^®^) against *Ctenocephalides felis* fleas was evaluated in two studies. Study A assessed the efficacy against existing fleas whereas study B assessed the efficacy against new infesting fleas. In study A, 12 dogs were allocated to the untreated group and 20 dogs to the treated group. All dogs were infested by 100 fleas each at Day −1, treated at Day 0 and flea combed at 2 h or at 6 h post treatment. In study B, 6 dogs were allocated to the untreated group and 10 to the treated group. They were infested with 100 fleas each on Days 2, 7, 14, 21 and 28. Fleas were removed and counted at 6 h post-infestation. Immediate and persistent efficacies were evaluated by counting fleas on the dogs. To evaluate induced mortality after exposure on dogs, fleas collected alive were placed in an insectarium for 24 h and assessed for viability. The immediate efficacy on dogs was significant at 6 h with 100%. The induced death of the fleas collected live from dogs 2 h after exposure was 99.7%. Concerning new infesting fleas, the observed efficacy at 6 h and the induced mortality were significantly different (*p* < 0.05) from the control at all time-points. At 6 h, the prophylactic efficacy was > 97% at Day 2 and Day 8 and > 90% at Day 14. The induced mortality after 6 h of exposure on dogs varied between 73.3% and 100% for the whole study.

## Abstract (fr)

La vitesse d’efficacité de l’afoxolaner (NexGard^®^) vis-à-vis de la puce *Ctenocephalides felis* a été évaluée dans deux études. L’étude A a évalué l’efficacité contre des puces existantes alors que l’étude B a évalué l’efficacité contre des puces nouvellement infestantes. Dans l’étude A, 12 chiens ont été répartis dans le groupe non traité et 20 chiens dans le groupe traité. Tous les chiens ont été infestés par 100 puces au jour −1, traités à J0 et peignés pour récolter les puces 2 heures ou 6 heures après traitement. Dans l’étude B, 6 chiens ont été répartis dans le groupe contrôle et 10 chiens dans le groupe traité. Ils ont été infestés avec 100 puces aux jours 2, 7, 14, 21 et 28. Les puces ont été collectées 6 heures après chaque infestation. Les efficacités immédiate et persistante ont été évaluées via le comptage des puces sur les chiens. Pour évaluer la mortalité induite après exposition sur les chiens, les puces collectées vivantes ont été placées en insectarium pendant 24 heures puis leur viabilité observée. L’efficacité immédiate était significative avec 100 % à 6 heures. La mortalité induite sur les puces collectées vivantes sur chiens à 2 heures a été de 99,7 %. Concernant les puces nouvellement infestantes, l’efficacité observée et la mortalité induite ont été significativement différente (*p* < 0,05) du contrôle à tous les comptages. À 6 heures, l’efficacité prophylactique était > 97 % à J2 et J8 et > 90 % à J14. La mortalité induite après une exposition de 6 heures sur les chiens variait de 73,3 à 100 % durant l’étude.

## Introduction

Fleas represent the most prevalent parasites in domestic carnivores [2, 17, 18]. The cat flea, *Ctenocephalides felis,* is the main flea species infesting both dogs and cats [8, 14]. In addition to causing discomfort to pets and their owners, cat fleas are associated with several diseases. Indeed *C. felis* is primarily responsible for flea bite allergy dermatitis (FAD) in dogs and cats [5, 7, 19, 20]. The cat flea is also the primary intermediate host of the tapeworm *Dipylidium caninum*, the common intestinal cestode of dogs and cats [9, 20]. *C. felis* can also transmit flea-borne spotted fever (*Rickettsia felis*), and some *Bartonella* species, such as *B. henselae,* the agent of Cat Scratch Disease [1, 13, 16]. Fleas are also vectors of some filarioids such as *Acanthocheilonema reconditum* infesting dogs in many parts of the world [4].

Although the use of insecticides such as fipronil, imidacloprid, selamectin and spinosad have revolutionised flea control in recent years, treatment and prevention of cat flea infestations remain a major concern for pet owners and veterinarians [2, 24]. The research continues for new flea control compounds that are effective, fast acting, long lasting, and easy to administer. Licensed products must reach standards of efficacy determined by pharmaceutical regulatory authorities worldwide (European Medicine Agency (EMA) in Europe, Environmental Protection Agency (EPA) or Food and Drug Administration (FDA) in USA). To meet EMA guidelines, the efficacy of a drug against fleas must reach at least 95%, 48 h post infestation for a given time-point in several controlled standard studies conducted [10].

Over the past decade speed of kill on fleas has been recognized as an increasingly important criterion of efficacy [2] prompting the evaluation of flea efficacy after shorter exposures than the ones required by regulatory agencies worldwide. The speed of kill is also an important criterion to estimate the risk reduction for transmission of vector-borne diseases, especially in regard to tick-borne pathogens [2, 17, 18].

Afoxolaner is a new insecticide/acaricide molecule from the isoxazoline family that acts on the insect *γ*-aminobutyric acid receptor (GABA) and glutamate receptors, inhibiting GABA & glutamate-regulated uptake of chloride ions, resulting in excess neuronal stimulation and death of the arthropod [12, 27]. Afoxolaner is the active ingredient of NexGard^®^, a recently approved oral ectoparasiticide product for dogs. NexGard^®^ administered to dogs has been demonstrated to provide an efficacy higher than 95% within 12 h against adult fleas (*Ctenocephalides felis* and *C. canis*) for at least 3 weeks, and within 24 h for at least 5 weeks [15]. The two studies presented here were intended to evaluate the killing activity of afoxolaner against adult *C. felis* fleas after short exposure times of 2–6 h on dogs, either on existing fleas (immediate or curative efficacy) or on new infesting fleas (prophylactic efficacy).

## Materials and methods

The two studies were parallel group design, randomised, single centre, controlled efficacy studies. Study A assessed the efficacy of afoxolaner against existing fleas, whereas study B assessed efficacy against new infesting fleas. In total, 48 dogs were included in the two studies, 32 in study A and 16 in study B. All 32 study A dogs and 16 study B dogs were infested with 100 (±5) unfed adult *C. felis* on Day −7, which were removed and counted on Day −6 for allocation purposes.

In study A, 32 mongrel dogs were randomly allocated to the untreated control group (12 dogs) or to the NexGard^®^ orally treated group (20 dogs). All dogs were infested by 100 fleas each at Day −1 and were treated at Day 0. Dogs were flea combed at 2 h (6 control and 10 treated dogs) or at 6 h (6 control and 10 treated dogs) post treatment.

In study B, 16 dogs were allocated to the negative untreated control group (*n* = 6) or to the NexGard^®^ treated group (*n* = 10).

The design of the two studies was in accordance with the World Association for the Advancement of Veterinary Parasitology (W.A.A.V.P.) guidelines for evaluating the efficacy of parasiticides for the treatment, prevention and control of flea and tick infestation on dogs and cats” [6]. The studies were authorized by both Merial and ClinVet ethics committees, and were conducted in respect of Good Clinical Practices as described in International Cooperation on Harmonisation of Technical Requirements for Registration of Veterinary Medicinal Products (VICH) guideline GL9 [10].

### Animals

The dogs were acclimated at the study facility for at least seven days before Day 0; they were clinically healthy as determined by a veterinarian on Day −7; they were older than 6 months, not clinically pregnant, and they had not been treated with a topical or systemic acaricide/insecticide within 12 weeks preceding Day 0. Study animals were kept individually in cages and no contact between dogs was possible. The animals were fed once a day. Food and water were provided in stainless steel bowls and the water was replenished at least twice daily.

At Day 0, dogs in the NexGard^®^ treated group were dosed once orally with the appropriate soft chew formulations containing afoxolaner, following approved label recommendations. The dogs were weighed on Day −1. As all treated dogs weighed between 10 and 25 kg, they received a 3 g chew containing 68 mg of afoxolaner. The dogs were observed hourly (±15 min) for 4 h after the last animal had been treated for possible adverse events.

### Flea infestations

A laboratory-bred European strain (ClinVet, Bloemfontein, South Africa) of *Ctenocephalides felis* was used for all infestations. For the evaluation of efficacy, each dog was infested with 100 (±5) unfed adult *C. felis* once on Day −1 in study A and on Days 2, 7, 14, 21 and 28 in study B.

### Flea counts and removal

Flea removal and counts on dogs were performed on Day 0 (study A) at 2 h (for 6 control and 10 treated dogs) and at 6 h (for 6 control and 10 treated dogs) in study A. In study B, flea removal and counts were performed on Days 2, 7, 14, 21 and 28, 6 h after each infestation. For combing, a fine-toothed flea comb was used to recover fleas present in the animal’s fur. The method of combing was by several strokes of the comb in each area of the animal, each time moving in the same direction following the pattern of the hair coat. Movement, from one part of the animal’s fur to the next was via strokes overlapping each other, so that no area of fur was missed. After completion of the combing procedure for all body areas, the whole procedure was repeated once more so that all areas were combed twice. The fleas were categorized in three categories as: live (i.e. normal behaving); moribund (i.e. dying fleas); and dead fleas.

### Flea assessments in insectarium

At all time-points in both studies, live collected fleas were placed in vials at the time of collection, identified with at least the dog’s identification, study number and date, and maintained in an insectarium for 24 h. They were held in an environmental chamber at ~75% relative humidity and 24–28 °C (±2 °C). These fleas were evaluated for viability on Day 1 (study A), and Days 3, 8, 15, 22 and 29 (study B).

### Effectiveness criteria

The primary criterion was the number of live fleas counted on the dog at the time of removal. It allowed the assessment of the killing efficacy observed on dogs, either immediate/curative or persistent/prophylactic.

A secondary criterion was assessment of the viability of live fleas kept in an insectarium for 24 h after collection off dogs compared to controls. This secondary criterion measures the afoxolaner-induced flea mortality after they have been removed off of dogs, and prevented any additional drug exposure. It allowed the assessment of induced mortality after a very short exposure to afoxolaner, i.e. the mortality rate after a given exposure time on treated dogs compared to control dogs, based on the 100 fleas deposited on each dog at each infestation.

### Statistical analysis

The observed efficacy on dogs against fleas counted after a short exposure (30 min, 2 h or 6 h) was calculated according to the following formula:

$$\mathrm{Efficacy}\;\left(\;\%\right)=100\times \left(m_{c}-m_{t}\right)/m_{c},$$

where

*m*_c_ = geometric or arithmetic mean of live fleas collected from the dogs in the negative control group at each collection time-point;

*m*_t_ = geometric or arithmetic mean of live fleas collected from the dogs in the administration group at each collection time-point.

The induced death rate was calculated for the administration groups according to the following formula:

$$\mathrm{Efficacy}\;\left(\%\right)=100\times \left(m_{c}-m_{t}\right)/m_{c},$$

*m*_c_ = geometric or arithmetic mean of live fleas in insectarium from the negative control group;

*m*_t_ = geometric or arithmetic mean of live fleas in insectarium from the treated group.

The number of live fleas observed per dog in the insectarium is related to the original 100 fleas put on each dog. The remaining live fleas after a certain period of time are placed in the insectarium and then assessed at 24 h. The comparison between treated and untreated dogs is linked to the 100 fleas put on all animals in both groups. Therefore, the induced death is a cumulative assessment of the killing efficacy (immediate and delayed) after a certain exposure time.

The untransformed flea count data were compared using a non-parametric Mann-Whitney U test. SAS^®^ Version 9.3 was used for all the statistical analyses. The level of significance of the formal tests was set at 5%, all tests were two sided.

## Results

In study A, no adverse events were noted.

In study B, one control dog presented a footpad laceration. No adverse event was related to the administration of afoxolaner.

Study A assessed the immediate curative efficacy on existing fleas (Table 1). The curative efficacy against fleas on dogs was significant after just 6 h with 100% effect. The induced flea mortality observed after 24 h in the insectarium was 99.7% for the fleas collected 2 h after exposure, which was significant versus the control (*p* = 0.00138).

**Table 1.** Immediate efficacy and induced mortality of afoxolaner against fleas after 2 h or 6 h of exposure on dogs.

	Immediate curative efficacy (Counts of live fleas removed from dogs*)				Induced mortality (Counts of live fleas after 24 h in insectarium)			
	Control group (*n* = 6)	Treated group (*n* = 10)			Control group (*n* = 6)	Treated group (*n* = 10)		
Day −1 flea infestation	Control mean	Arithmetic mean	Geometric mean (Eff%)	*p*-value	Control mean	Arithmetic mean	Geometric mean (Eff%)	*p*-value
Day 0 + 2 h	75.5 (57–89)	74.5	70.5 (5.5)	>0.05	69.5 (47–86)	0.6	0.2 (99.7)	0.00138*
Day 0 + 6 h	67.8 (40–88)	0	0 (100)	0.0138*	62.7 (28–86)	/	/	/

* The live fleas removed from dogs were put in the insectarium for 24 h. Induced mortality was evaluated as the survival of fleas measured in the insectarium after 24 h.

Day of treatment (Day 0). Time (in hours) after treatment (Day 0).

Study B assessed the prophylactic efficacy after only a short exposure time on dogs against new infesting fleas during the month after treatment.

The persistent efficacy observed on Days 7, 14, 21, 28 after 6 h of exposure on treated dogs varied between 29% and 96.7% based on arithmetic means with the highest percentage of efficacy obtained on Day 7 and the lowest at Day 28 (Table 2). The live flea counts differed significantly (*p* < 0.05) between control and treated groups at all assessment time-points.

**Table 2:** Observed prophylactic efficacy of afoxolaner and induced mortality of fleas after 6 h of exposure on dogs.

	Observed efficacy on dogs (Live fleas removed from dogs)				Induced mortality (Live fleas counted after 24 h in insectarium)			
	Control group (*n* = 6)	Treated group (*n* = 10)			Control group (*n* = 6)	Treated group (*n* = 10)		
Infestation day	Arithmetic mean	Arithmetic mean (Eff%)	Geometric mean^1^ (Eff%)	*p*-value^2^	Arithmetic mean	Arithmetic mean (Eff%)	Geometric mean^1^ (Eff%)	*p*-value^2^
Day 2	94.2	4.2 (95.5)	1.7 (98.2)	0.0012*	93.7	0.6 (99.4)	0.3 (99.7)	0.0006*
Day 7	74.7	2.5 (96.7)	1.8 (97.5)	0.0013*	64.0	0.0 (100.0)	0.0 (100.0)	0.0008*
Day 14	80.2	8.1 (89.9)	7.1 (90.9)	0.0014*	50.7	0.0 (100.0)	0.0 (100.0)	0.0012*
Day 21	71.8	27.4 (61.9)	22.4 (68.3)	0.0056*	59.3	2.2 (96.3)	1.8 (96.7)	0.0012*
Day 28	89.7	63.7 (29.0)	63.2 (29.2)	0.0014*	72.8	25.8 (64.6)	18.5 (73.3)	0.0048*

1 Computed as the anti-logarithm of the arithmetic mean of the log-counts and then subtracting 1.

2 (Two-sided) Probability value associated with the comparison of the population means of the two treatment groups.

* Significant difference.

The mortality observed in the insectarium from fleas collected live after 6 h of exposure on dogs varied between 73.3% and 100% based on geometric means with the highest percentage efficacy obtained on Days 7 and 14. The flea survival differed significantly (*p* < 0.05) from the control on all assessments.

## Discussion

Afoxolaner is a new systemic insecticide and acaricide. It was therefore important to assess its speed of action on fleas. These studies demonstrated that afoxolaner completely killed existing fleas on dogs in 6 h and induced a significant mortality after just 2 h of exposure. It induced the mortality of 97.6 to 100% of new infesting fleas after 6 h of exposure on dogs until Day 21. This property can be related to both the quick absorption and plasma peak [14], but also to the volume of blood ingested by the fleas. Afoxolaner is detected in plasma 20–30 min after oral administration and reaches its peak in 2–4 h. The frequency of flea bites is also a factor explaining the quick intoxication of fleas, which will ingest the insecticide present in the blood [25]. New infesting fleas may bite as soon as 20 min after infesting their host and almost all fleas feed in the first 2 h. The number of flea bites per day is unknown. Fleas can stop their meal when they are disturbed and it is possible that they may bite more often but without ingesting a sufficient volume. The curative efficacy and its speed may then be related to both the number of bites, the volume ingested by fleas, and by the number of fleas that will bite in a certain time on the host. The 5.5% curative efficacy observed at 2 h may be due to a low number of fleas having bitten, but when looking at the induced mortality of 99.7% of these fleas, it is more probably related to the volume of drug ingested. After 6 h of exposure to afoxolaner, all existing fleas were dead, which would mean that fleas having ingested more drug, died quicker.

Until now, the majority of anti-flea treatments were based on topical spot-on applications [2, 24], with the active molecules diffusing onto the skin and acting by contact with the arthropods (e.g. dinotefuran, fipronil, imidacloprid) or being absorbed to act systemically (i.e. selamectin). Recently, oral formulations appeared on the market, based on either nitenpyram [21], spinosad [3, 23, 26], afoxolaner [27] or fluralaner [22]. They provide a fast curative efficacy on existing flea infestation. Nitenpyram kills existing fleas on dogs between 15 min and 24 h (3–8 h in some papers) but it has no persistent activity with only about 24–48 h of anti-flea efficacy [11, 21]. Spinosad oral administration provides a curative efficacy, starting to kill fleas within 30 min and killing >95% in about 4 h, combined with up to 4 weeks preventive efficacy on new flea infestations when counted at 48 h after weekly flea infestations [23, 26]. With afoxolaner, the prophylactic efficacy and the induced mortality were significant during the full month after 6 h of exposure, with an induced mortality over 96.7% through Day 21. The decrease in observed efficacy at 6 h on dogs the third and the fourth week may be related to a lower concentration circulating in the blood. However, the dose ingested was still enough to induce the death of the fleas during the month as shown by the mortality rate in the insectarium and by the on dog efficacy studies published at 12 h and 24 h counts [12]. The new oral insecticide-acaricide afoxolaner is able to induce the death of fleas after a short exposure time on treated dogs.

## Conflict of interest

This clinical study was funded by Merial S.A.S., 29 avenue Tony Garnier, 69007 Lyon of which Frédéric Beugnet and Lénaïg Halos are employees. ClinVet, of which Josephus Fourie and Christa deVos are employees, is an independent, South African, Contract Research Organisation contracted to conduct the study. All authors voluntarily publish this article and have no personal interest in these studies other than publishing the scientific findings that they have been involved in via planning, initiating, monitoring and conducting the investigations and analysing the results. This document is provided for scientific purposes only. Any reference to a brand or trademark herein is for informational purposes only and is not intended for a commercial purpose or to dilute the rights of the respective owner(s) of the brand(s) or trademark(s). NexGard^®^ is a registered trademark of Merial. All other marks are the property of their respective owners.

## References

[1] Azad A, Radulovic S, Higgins JA, Noden BH, Troyer JM. 1997 Flea-borne rickettsioses: ecologic considerations. Emerging Infectious Diseases, 3, 319–327 9284376 10.3201/eid0303.970308 PMC2627639

[2] Beugnet F, Franc M. 2012 Insecticide and acaricide molecules and/or combinations to prevent pet infestation by ectoparasites. Trends in Parasitology, 28, 267–279 22627116 10.1016/j.pt.2012.04.004

[3] Blagburn BL, Young DR, Moran C, Meyer JA, Leigh-Heffron A, Paarlberg T, Zimmermann AG, Mowrey D, Wiseman S, Snyde DE. 2010 Effects of orally administered spinosad (Comfortis^®^) in dogs on adult and immature stages of the cat flea (*Ctenocephalides felis*). Veterinary Parasitology, 168, 312–317 20045256 10.1016/j.vetpar.2009.11.023

[4] Brianti E, Gaglio G, Napoli E, Giannetto S, Dantas-Torres F, Bain O, Otranto D. 2012 New insights into the ecology and biology of *Acanthocheilonema reconditum* (Grassi, 1889) causing canine subcutaneous filariosis. Parasitology, 139, 530–536 22336052 10.1017/S0031182011002198

[5] Carlotti DN, Costargent F. 1994 Analysis of positive skin tests in 449 dogs with allergic dermatitis. European Journal Companion Animal Practice, 4, 42–59

[6] Dobson P, Tinembart O, Fisch RD, Junquera P. 2000 Efficacy of nitenpyram as a systemic flea adulticide in dogs and cats. Veterinary Record, 147, 709–713 11140929

[7] Dryden MW, Blakemore JC. 1989 A review of flea allergy dermatitis in the dog and cat. Companion Animal Practice, 19, 10–17

[8] Dryden MW, Rust MK. 1984 The cat flea: biology, ecology and control. Veterinary Parasitology, 52, 1–19 8030176 10.1016/0304-4017(94)90031-0

[9] Dunn AM. 1978 Cestoda, in Veterinary Helminthology, 2nd edn Heinemann: London

[10] EMEA. 2000 VICH Topic GL9 (GCP). Guideline on Good Clinical Practices. The European Agency for the Evaluation of Medicinal Products (EMWA/CVMP/VICH/595/98-Final). http://www.ema.europa.eu/docs/en_GB/document_library/Scientific_guideline/2009/10/WC500004343.pdf)

[11] Franc M, Bouhsira E. 2009 Evaluation of speed and duration of efficacy of spinosad tablets for treatment and control of *Ctenocephalides canis* (Siphonaptera: Pulicidae) infestations in dogs. Parasite, 16, 125–128 19585890 10.1051/parasite/2009162125

[12] Hunter JS III, Dumont P, Chester TS, Young DR, Fourie JJ, Larsen DL. 2014 Evaluation of the curative and preventive efficacy of a single oral administration of afoxolaner against cat flea *Ctenocephalides felis* infestations on dogs. Veterinary Parasitology, 201, 207–211 24629423 10.1016/j.vetpar.2014.02.024

[13] Just FT, Gilles J, Pradel I, Pfalzer S, Lengauer H, Hellmann K, Pfister K. 2008 Molecular evidence of *Bartonella* spp. in cat and dog fleas from Germany and France. Zoonoses Public Health, 55, 514–520 18489542 10.1111/j.1863-2378.2008.01131.x

[14] Letendre L, Harriman J, Huang R, Kvaternick V, Drag M, Larsen DL. 2014 The intravenous and oral pharmacokinetics of afoxolaner, a novel isoxazoline, used as a monthly chewable antiparasitic for dogs. Veterinary Parasitology, 201, 190–197 24685320 10.1016/j.vetpar.2014.02.021

[15] Marchiondo AA, Holdsworth PA, Fourie LJ, Rugg D, Kellmann K, Snyder DE, Dryden MW. 2013 World Association for the Advancement of Veterinary Parasitology (W.A.A.V.P.) second edition: guidelines for evaluating the efficacy of parasiticides for the treatment, prevention and control of flea and tick infestations on dogs and cats. Veterinary Parasitology, 194, 84–97 23741753 10.1016/j.vetpar.2013.02.003

[16] Orloski KA, Lathrop SL. 2003 Plague: a veterinary perspective. Vet. Med. Today: Zoonosis Update. Journal of the American Veterinary Medical Association, 222, 444–448 12597416 10.2460/javma.2003.222.444

[17] Otranto D, Dantas-Torres F, Breitschwerdt EB. 2009 Managing canine vector-borne diseases of zoonotic concern: part one. Trends in Parasitology, 25, 157–163 19269898 10.1016/j.pt.2009.01.003

[18] Otranto D, Dantas-Torres F, Breitschwerdt EB. 2009 Managing canine vector-borne diseases of zoonotic concern: part two. Trends in Parasitology, 25, 228–235 19346164 10.1016/j.pt.2009.02.005

[19] Plant JD. 1991 Recognizing the manifestations of flea allergy in cats. Veterinary Medicine, 10, 482–486

[20] Pugh RE. 1987 Effects on the development of *Dipylidium caninum* and on the host reaction to this parasite in the adult flea (*Ctenocephalides felis felis*). Parasitology Research, 73, 171–177 3575292 10.1007/BF00536475

[21] Robertson-Plouch C, Baker KA, Hozak RR, Zimmermann AG, Parks SC. 2008 Clinical field study of the safety and efficacy of spinosad chewable tablets for controlling fleas on dogs. Veterinary Therapeutics, 9, 26–36 18415944

[22] Rohdich N, Roepke R, Zschiesche E. 2014 A randomized, blinded, controlled and multi-centered field study comparing the efficacy and safety of bravecto™ (fluralaner) against frontline™ (fipronil) in flea- and tick-infested dogs. Parasites & Vectors, 7, 83 24593931 10.1186/1756-3305-7-83 PMC3975895

[23] Ross DH, Arther RG, von Simson C, Doyle V, Dryden MW. 2012 Evaluation of the efficacy of topically administered imidacloprid + pyriproxyfen and orally administered spinosad against cat fleas (*Ctenocephalides felis*): Impact of treated dogs on flea life stages in a simulated home environment. Parasites & Vectors, 5, 192 22958307 10.1186/1756-3305-5-192 PMC3514229

[24] Rust MK. 2005 Advances in the control of *Ctenocephalides felis* (cat flea) on cats and dogs. Trends in Parasitology, 21, 232–236 15837612 10.1016/j.pt.2005.03.010

[25] Rust MK, Dryden MW. 1997 The biology, ecology and management of the cat flea. Annual Review Entomology, 42, 451–473 10.1146/annurev.ento.42.1.451 9017899

[26] Schenker R, Tinembart O, Hubert-Droz E, Cavaliero T, Yerly B. 2003 Comparative speed of kill between nitenpyram, fipronil, imidacloprid, selamectin and cythioate against adult *Ctenocephalides felis* (Bouché) on cats and dogs. Veterinary Parasitology, 112, 249–254 12591200 10.1016/s0304-4017(02)00425-9

[27] Shoop WL, Hartline EJ, Gould BR, Waddell ME, McDowell RG, Kinney JB, Lahm GP, Long JK, Xu M, Wagerle T, Jones GS, Dietrich RF, Cordova D, Schroeder ME, Rhoades DF, Benner EA, Confalone PN. 2014 Discovery and mode of action of afoxolaner, a new isoxazoline parasiticide for dogs. Veterinary Parasitology, 201, 179–189 24631502 10.1016/j.vetpar.2014.02.020

